# Metabolic adaptations in antibiotic-resistant *Staphylococcus aureus*: understanding resistance mechanisms and enhancing antibiotic efficacy

**DOI:** 10.1128/spectrum.00020-26

**Published:** 2026-06-29

**Authors:** Zhiyu Pan, Wan Zhang, Zhuo Ying Cao, Liting Cai, Yubin Su, Jiao Fei

**Affiliations:** 1 Department of Blood Transfusion, The First Affiliated Hospital, Jinan University 47885, Guangzhou, China; 2 The Clinical Medical Laboratory Center, The First Affiliated Hospital, Jinan University 47885, Guangzhou, China; 3 MOE Key Laboratory of Viral Pathogenesis & Infection Prevention and Control (Jinan University) and National Engineering Research Center of Genetic Medicine, MOE Key Laboratory of Tumor Molecular Biology, Guangdong Provincial Key Laboratory of Bioengineering Medicine, Department of Immunology and Microbiology, Institute of Medical Microbiology, College of Life Science and Technology, Jinan University 47885, Guangzhou, China; 4 State Key Laboratory of Tropical Oceanography, South China Sea Institute of Oceanology, Chinese Academy of Sciences, Guangzhou, China; The Pennsylvania State University, University Park, Pennsylvania, USA

**Keywords:** ciprofloxacin/cefazolin-resistant *S. aureus*, antibiotic resistance mechanism, central carbon metabolism, metabolic regulation

## Abstract

The global rise of antibiotic-resistant *Staphylococcus aureus* represents a serious clinical challenge, contributing substantially to the burden of antimicrobial resistance. This study explores the metabolic mechanisms of antibiotic-resistant *S. aureus*. Using LC-MS/MS metabolomics, we analyzed laboratory-evolved strains derived from the Newman strain: ciprofloxacin-resistant (R-CIP), cefazolin-resistant (R-CEF), and dual-resistant (R-CC). Metabolomic profiling revealed 41, 57, and 74 differentially abundant metabolites in R-CIP, R-CEF, and R-CC, respectively, with 21 common to all strains. Pathway analysis identified 12, 9, and 10 significantly perturbed pathways in R-CIP, R-CEF, and R-CC, respectively. Three pathways—glycine, serine, and threonine metabolism; alanine, aspartate, and glutamate metabolism; and taurine and hypotaurine metabolism—were consistently dysregulated across all resistant strains. R-CIP uniquely altered butanoate metabolism, while R-CC specifically changed glutamine and purine metabolism. iPath analysis further highlighted suppressed central carbon and energy metabolism as a shared feature of all resistant strains, accompanied by reduced pyruvate dehydrogenase and TCA cycle enzyme activities, as well as decreased cellular NADH, ATP, and reactive oxygen species levels. Supplementation with pyruvate, citrate, or fumarate significantly enhanced the bactericidal effects of ciprofloxacin and cefazolin. These findings demonstrate that *S. aureus* acquires resistance to ciprofloxacin and cefazolin through downregulation of central carbon and energy metabolism. Exogenous metabolites can potentiate antibiotic efficacy, offering insights into metabolism-targeted strategies to combat antibiotic-resistant *S. aureus*.

**IMPORTANCE:**

The global rise of antibiotic-resistant *Staphylococcus aureus* poses a significant threat to public health, highlighting the urgent need for novel therapeutic strategies. Current understanding of antibiotic resistance mechanisms, particularly at the metabolic level, remains incomplete. This study investigates the metabolic alterations of resistance in *S. aureus* by analyzing laboratory-evolved strains resistant to ciprofloxacin (R-CIP), cefazolin (R-CEF), and both (R-CC). Through metabolomic profiling, we identified suppressed central carbon and energy metabolism as a shared resistance mechanism, with strain-specific adaptations further complicating the resistance landscape. Notably, supplementation with key metabolites like pyruvate, citrate, and fumarate enhanced antibiotic efficacy, suggesting a promising strategy to combat resistance. These findings provide critical insights into the metabolic vulnerabilities of resistant *S. aureus*, offering a novel avenue for the development of metabolism-targeted therapies to address the growing challenge of antibiotic resistance.

## INTRODUCTION

Antibiotics are a cornerstone of modern medicine. They remain the primary treatment for bacterial infections and are indispensable in healthcare, agriculture, and food production (1, 2). However, the effectiveness of these essential drugs is increasingly undermined by the rapid spread of antibiotic resistance. While resistance emerges predictably under selective pressure, its accelerated global dissemination now threatens public health and food security worldwide (3, 4). This crisis is driven by the overprescription and misuse of antibiotics in human medicine, gaps in infection prevention and control, and the extensive non-therapeutic use of these drugs in livestock farming (5–8). Compounding the problem are the lack of rapid diagnostic tools and the dwindling pipeline of new antibacterial agents, which together limit our ability to combat resistant infections effectively (9, 10).

*Staphylococcus aureus* remains a leading cause of both community- and hospital-acquired infections, ranging from mild skin conditions to life-threatening systemic diseases (11). In its 2024 priority pathogens list, the World Health Organization classified *S. aureus* as a “high-priority” bacterium requiring urgent research and development attention, reflecting its rapid spread, increasing resistance to last-resort agents, and high transmissibility—especially in healthcare environments (12, 13). One of the most clinically significant resistance mechanisms in *S. aureus* is the acquisition of the staphylococcal cassette chromosome *mec* (SCC*mec*), a mobile genetic element carrying the *mecA* gene. This gene encodes the alternative penicillin-binding protein PBP2a, which exhibits low affinity for most β-lactams, conferring resistance to methicillin and related antibiotics. Strains carrying *mecA* are designated methicillin-resistant *S. aureus* (MRSA) (14–18). The potent bactericidal activity of the fifth-generation cephalosporin ceftaroline fosamil against Gram-positive bacteria, including MRSA, is mediated by its high-affinity binding to PBP2a, thereby inhibiting cell wall synthesis (19). Despite its potency, high-level ceftaroline resistance in MRSA has emerged. Mechanistic studies indicate that this resistance is primarily driven by two adjacent substitutions (Y446N and E447K) within the antibiotic-binding pocket of the PBP2a transpeptidase domain, which compromise ceftaroline binding (20). Beyond β-lactam resistance, mutations in fluoroquinolone targets—such as *gyrA*, *gyrB*, and *parC*—lower drug affinity and allow survival under treatment (21). While such specific molecular mechanisms are well defined, the overall process by which antibiotics kill *S. aureus*—and how resistant strains evade killing—involves a broader network of adaptive responses. These include stress-response activation, biofilm formation, and metabolic reprogramming, all of which can work together to enhance bacterial survival (22). Despite decades of research, drug-resistant *S. aureus*, remains a pressing and formidable challenge in clinical settings worldwide. This underscores the critical need for novel therapeutic strategies that can overcome or bypass these multifactorial resistance mechanisms.

Recent advances in high-throughput metabolomics have provided a powerful platform for investigating the metabolic basis of bacterial antibiotic resistance (23, 24). A bacterium’s susceptibility to antibiotics is closely linked to its metabolic state, which is continuously shaped by physiological and environmental conditions (25, 26). Antibiotic exposure perturbs this state, and comparative metabolomic profiling of drug-sensitive and drug-resistant strains can pinpoint key metabolic pathways underlying resistance mechanisms (27–30). For example, targeted metabolomics is applied to study methicillin-susceptible (MSSA) and methicillin-resistant *S. aureus* (MRSA) strains under sublethal doses of β-lactam, aminoglycoside, and quinolone antibiotics. This work identified a set of both common and antibiotic-specific metabolic perturbations. Notably, upon β-lactam exposure, key pathways—including pyrimidine, amino acid, and purine metabolism are prominently altered (31). Building on such insights, a promising strategy known as “metabolic reprogramming” has emerged. This approach seeks to resensitize resistant bacteria by supplying specific exogenous metabolites, thereby shifting their internal metabolic state back toward antibiotic susceptibility (32–36). Previous investigations revealed that MRSA employs metabolic reprogramming as a key strategy for chronic lung colonization. This host adaptation is marked by specific changes, such as altered tricarboxylic acid cycle activity and fumarate-mediated biofilm production. They further indicate that modulating these metabolic pathways with targeted metabolites could potentially reverse antibiotic resistance and disrupt persistent infection (37). A recent study shows that docosahexaenoic acid (DHA) can reverse MRSA resistance to β-lactam antibiotics through metabolic reprogramming. DHA disrupts both lipid and iron metabolism, triggering ferroptosis-like cell death characterized by lipid peroxidation and iron homeostasis dysregulation, which in turn restores and enhances the efficacy of β-lactam antibiotics (38).

In our previous study, we compared the metabolomes of artificially selected penicillin- and gentamycin-resistant *S. aureus* strains with those of susceptible counterparts. This analysis revealed that alterations in central carbon and energy metabolism, as well as in arginine biosynthesis, contribute meaningfully to drug resistance in this pathogen (39). To test whether these metabolic adaptations reflect a broader resistance mechanism in *S. aureus*, we extended the study using the well-characterized Newman strain. Here, we focused on two clinically relevant antibiotics: ciprofloxacin (CIP) and cefazolin (CEF). Through serial passaging under antibiotic pressure, we generated derivatives resistant to CIP (R-CIP), to CEF (R-CEF), and to both antibiotics simultaneously (R-CC). We then applied liquid chromatography–tandem mass spectrometry (LC-MS/MS) to profile the metabolomes of these strains alongside the parental, susceptible isolate, aiming to identify both shared and strain-specific metabolic changes.

We propose that *S. aureus* adapts to antibiotic pressure through systemic suppression of central carbon metabolism (glycolysis and TCA cycle), effectively lowering metabolic activity to enter a persistent, low-energy state that promotes tolerance and subsequent resistance development.

## MATERIALS AND METHODS

### Bacterial strain and culture conditions

*S. aureus* Newman was kindly provided by Professor Xuesong Sun, Jinan University. As previously reported (40), single colonies were incubated in Luria-Bertani (LB) medium at 37°C for 12 h with 220 rpm shaking. The overnight cultures were then transferred in fresh medium containing 1/2 minimum inhibitory concentration (MIC) of CIP or CEF at a 1:100 v/v ratio. Through stepwise increases in antibiotic concentration and serial passage over multiple generations, we induced and screened for strains of *S. aureus* resistant to CEF and CIP. The two resistant strains ultimately obtained were CEF-resistant strain (R-CEF, MIC increased 128-fold) and CIP-resistant strain (R-CIP, MIC increased 128-fold). Subsequently, the overnight culture of R-CIP was transferred to fresh medium containing 1/2 MIC of CEF. Similarly, a stepwise increase in antibiotic concentration and multi-generational culture were performed until the R-CC strain, concurrently resistant to both antibiotics, was obtained. The aforementioned resistant strains and sensitive control strains were inoculated into fresh medium and incubated with shaking at 37°C until reaching the mid-logarithmic growth phase (OD_600_ = 1.0). Subsequently, the bacterial cultures were centrifuged at 8,000 × *g* for 5 min to harvest the cell pellets. The pellets were washed three times with sterile saline and either resuspended in buffer or frozen in liquid nitrogen for further assays.

### MIC measurement and growth curve assay

Briefly, the MIC values of R-CEF, R-CIP, and R-CC for various antibiotics were measured based on the Clinical and Laboratory Standards Institute guidelines (41). Overnight cultures were diluted in fresh Mueller-Hinton Broth (MHB) at a ratio of 1:100 and then incubated at 37°C with 220 rpm shaking to an OD_600_ of 0.5. Antibiotics were serially diluted twofold with MHB medium, and then 90 μL was added to 96-well plates, and then 10^5^ colony-forming unit (CFU) bacteria were added to the 96-well plate. Finally, the 96-well plate was shaken several times and then placed at 37°C for 20−24 h of incubation. The lowest concentration of MIC that inhibited visual growth was recorded. As for the growth curve assay, cells were measured at 0, 2, 4, 6, 8, 10, 12, 14, 16 h for OD_600_ values. At least three biological replicates of the above experiment were performed.

### Metabolomic sample preparation and LC-MS/MS analysis

Briefly, metabolomic samples were prepared and analyzed, as previously described (39). The bacterial culture with an OD_600_ of 1.0 was collected by centrifugation at 8,000 × *g* for 5 min. The cell pellets were washed three times with sterile saline and immediately quenched with liquid nitrogen. Then, 1 mL of cold extract (acetonitrile:methanol:water = 2:2:1, v/v/v, containing ribitol internal standard 2 µg/mL) was added to the bacterial samples, and the samples were crushed by a 16-channel ultrasonic crusher in an ice-water bath, with a break of 5 s and a pause of 5 s, for a total of 15 min, and then incubated at 20℃ for 1 h; the supernatant was separated by centrifugation at a low temperature of 12,000 × *g* for 15 min, and then concentrated in vacuum. Subsequently, the dried samples were re-dissolved in acetonitrile and then centrifuged at 12,000 g at 4°C to collect the supernatant for LC-MS/MS analysis. The samples were analyzed by chromatographic separation using a Thermo Scientific UltiMate 3000 Rapid Separation Liquid Phase System, and the software Xcalibur 4.0.27 (Thermo) was used to control the mass spectrometer for primary and secondary mass spectrometry data acquisition.

### Metabolomic data analysis

The data were normalized for internal standard data and filled with missing data using MATLAB R2020a software. Total amount and interquartile range corrections were used to calibrate the data. The standardized data were analyzed by Mann-Whitney rank sum test (Mann-Whitney U test) by IBM SPSS Statistics 22, and metabolites with significant differences (*P* < 0.05) were selected for following analyses. MetaboAnalyst 5.0 was used for KEGG pathway enrichment of differential metabolites. Principal component analysis and orthogonal partial least squares discriminant analysis (OPLS-DA) models were implemented by SIMCA-P 12.0 software (version 12; Umetrics, Umea, Sweden). Interactive path (iPath) analysis was performed on iPath3.0 (https://pathways.embl.de/).

### Measurement of ATP

ATP was determined using the BacTiter-GloTM Microbial Cell Viability Assay (BacTiter-GloTM Microbial Cell Viability Assay, G8231, Promega, Madison, WI, United States). Briefly, bacteria grown to OD_600_ = 1.0 were collected by centrifugation. The precipitate was resuspended with phosphate buffer solution to OD_600_ = 0.2, and then equal volumes of samples and kit reagents were mixed, incubated in the dark at 37°C for 5 min, followed by fluorescence measurement. Luminescence was measured by the Victor X5 multimode microplate reader (Biotek, Synergy HT, Vermont, USA).

### NADH measurement

NADH was measured as described previously (39). In brief, the overnight bacterial culture was transferred to fresh liquid medium. When the bacteria had grown to an OD_600_ of 1.0, the cells were collected by centrifugation at 8,000 × *g* for 5 min. The cell pellet was washed three times with sterile saline and then retained. Bacterial intracellular NADH content was measured using a NADH reagent kit (Beyotime, S0175, China). According to the manufacturer’s instructions, the NADH extract was added to the collected bacteria and then sonicated in an ice water bath (180 W, 2-s pulse, 3-s pause, total duration of 5 min), and incubated at 60°C for 5 min. The opposite extraction buffer (NADH extraction buffer was added for detection of NAD^+^) was added to neutralize the extracts. Following vortexing briefly, the neutralized extracts were centrifuged at 14,000 × *g* for 5 min, and the supernatants were collected. Subsequently, the samples to be tested and the kit working solution were mixed proportionally and incubated at room temperature for 1 h. The fluorescence value of OD_450_ was recorded.

### Enzyme activity assay

Pyruvate dehydrogenase (PDH), isocitrate dehydrogenase (ICDH), α-ketoglutarate dehydrogenase (OGDH), succinate dehydrogenase (SDH), and malate dehydrogenase (MDH) were measured, as previously described (42). The collected bacterial precipitates were resuspended in PBS and then added and incubated with 10 μL of 1,200 U/mL lysostaphin for 5 min to help lyse the cell wall, then sonicated on an ice water bath to completely break up the cell walls (180 W, 2-s pulse, 3-s pause). After centrifugation at 4°C and 12,000 × *g* for 10 min, the supernatant was collected. The protein concentration of the supernatant was determined using the BCA Protein Concentration Assay Kit (Beyotime, P0009, China), and then the enzyme activity was measured using 100 μg of protein. For PDH and OGDH measurement, the reaction mixture contained 0.15 mM 3-(4,5-dimethyl-2-thiazolyl)-2,5-diphenyl-2H-tetrazolium bromide (MTT), 2.5 mM MgCl_2_, 0.5 mM phenazine methosulfate (PMS), 0.2 mM thiamine PPi (TPP), 50 mM potassium phosphate buffer, and 80 mM sodium pyruvate/alpha-ketoglutaric acid potassium salt, with water added to 200 μL. For SDH and MDH measurement, the reaction mixture contained 0.15 mM MTT, 2.5 mM MgCl_2_, 1 mM PMS, 50 mM potassium phosphate buffer, and 80 mM sodium succinate/sodium malate, with water added to 200 μL. For ICDH measurement, the reaction mixture contained 0.15 mM MTT, 2.5 mM MgCl_2_, 0.5 mM PMS, 1 M Tris-HCl, and 70 mM sodium isocitrate, with water added to 200 μL. The reaction mixtures were mixed well and then incubated at 37°C under light protection for 30 min for MDH and OGDH, and 15 min for ICDH, SDH, and PDH. Absorbance values were recorded at OD_566_. Citrate synthase (CS) activity was measured using a commercial assay kit (Solarbio, BC1060, China) according to the manufacturer’s instructions. The reaction was monitored by recording the absorbance at OD₄₁₂.

### Antibiotic bactericidal assay

A single bacterial colony was inoculated into LB broth and cultured overnight at 37°C with shaking at 220 rpm. The bacteria were harvested by centrifugation at 8,000 × *g* for 5 min and washed three times with sterile 0.85% saline. After discarding the final saline wash, the pellet was resuspended in M9 medium with 10 mM NaAc, 1 mM MgSO_4_, and 0.1 mM CaCl_2_ to an OD_600_ of 0.2 (39). Following the addition of relevant metabolites and/or antibiotics, the samples were incubated at 37°C with shaking at 220 rpm for 6 h. After incubation, 100 μL of culture was serially diluted, and 10 μL of each dilution was spotted onto LB agar plates. The plates were incubated statically at 37°C for 16 h before bacterial enumeration. The percentage of bacterial survival was calculated by dividing the colony-forming unit of the treated sample by that of the untreated control and multiplying by 100. All experiments were performed with three independent biological replicates.

### Measurement of ROS

As described previously (38), intracellular reactive oxygen species levels were measured using the fluorescent probe Mcarboxy-H2DCFDA (Sigma-Aldrich, USA). Bacterial cells were harvested, washed, and resuspended in pre-warmed PBS to an optical density (OD₆₀₀) of 0.2. The bacterial suspension was then incubated with 10 μL of Mcarboxy-H2DCFDA in the dark at 37°C for 1 h. After incubation, fluorescence was measured using a microplate reader with excitation and emission wavelengths set at 495 and 525 nm, respectively.

### Hemolysis test

The hemolytic activity of sensitive and resistant strains against red blood cells was evaluated using the erythrocyte hemolysis assay. The brief procedure is as follows: fresh mouse blood was collected and anticoagulated with heparin sodium, then centrifuged at 3,000 × *g* for 15 min to remove the supernatant. The red blood cells were washed three times with saline until the supernatant became clear, and the packed red blood cells were resuspended in saline to prepare a 2% red blood cell suspension, which was stored at 4°C for later use. After overnight culture, the experimental bacterial strains were transferred to fresh LB medium and grown to the logarithmic phase, then centrifuged at 8,000 × *g* for 3 min to collect the bacterial cells, which were resuspended in saline and adjusted to an OD_600_ of 1.0. In a 96-well plate, 100 μL of the red blood cell suspension was mixed with an equal volume of bacterial suspension, using double-distilled water (ddH_2_O; positive control) as references. After incubation at 37°C for 2 h, the supernatant was collected by centrifugation, and the absorbance at 576 nm was measured using a microplate reader. The hemolysis rate was calculated according to the formula: (OD value of sample well − OD value of negative control well) / (OD value of positive control well − OD value of negative control well). Three biological replicates were set up for each experiment.

## RESULTS

### Phenotype of antibiotic-resistant bacteria

**Fig 1:**
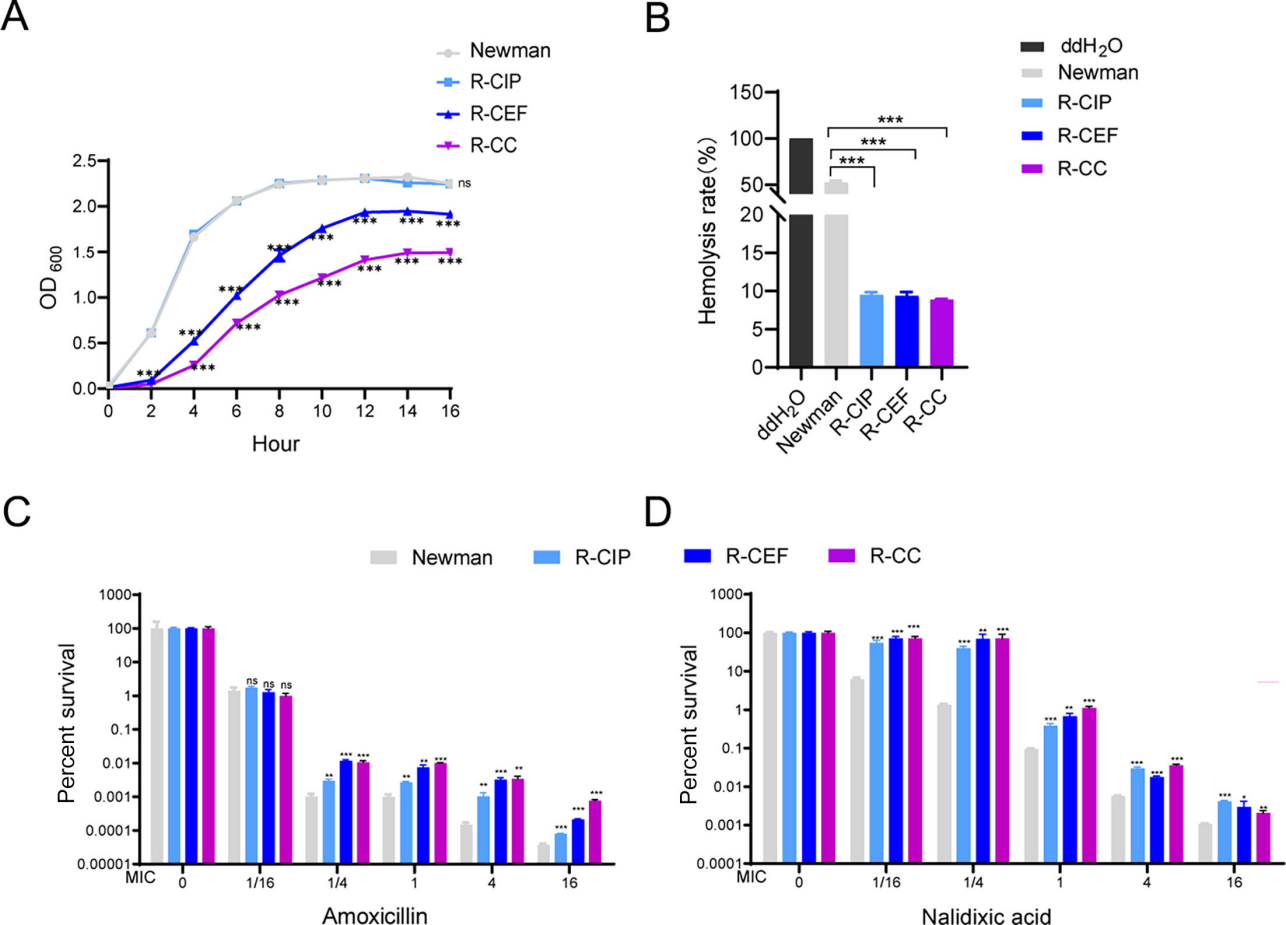
Antimicrobial resistance phenotype of bacteria. (**A**) Growth curve. (**B**) Hemolysis test of drug-resistant bacteria. ddH_2_O was set as the positive control. (**C and D**) Bacterial survival at different concentrations of amoxicillin or nalidixic acid. The results were shown as mean ± SEM, and the significant difference was determined by two-tailed Student *t* test, ns indicated no significant difference, *P < 0.05, **P < 0.01, ***P < 0.001.

Serial passaging of *S. aureus* Newman in the presence of sub-MIC (1/2 × MIC) concentrations of CIP or CEF produced resistant derivatives, designated R-CIP and R-CEF, respectively. Subsequent passaging of R-CIP under CEF pressure generated a dual-resistant strain, R-CC; a parallel passage control was maintained without antibiotic selection. Microdilution assays showed that R-CIP and R-CEF each exhibited a 128-fold increase in MIC for their respective selective antibiotics—reaching 40 μg/mL for CIP and 80 μg/mL for CEF—compared with the control strain; R-CC displayed a comparably elevated MIC (128-fold) to both CIP and CEF (Table 1). Growth-curve analysis revealed that R-CEF and R-CC grew significantly more slowly than the control, with the difference persisting beyond 16 h; growth inhibition was especially pronounced in R-CC. In contrast, R-CIP showed no significant difference in growth rate relative to the susceptible strain (Fig. 1A). We performed whole-genome sequencing on the four bacterial strains. Compared with the susceptible strain, R-CIP, R-CEF, and R-CC harbored 8, 6, and 20 non-synonymous or high-impact mutations, respectively (Tables S1 to S3). In R-CIP, key mutations with high predicted impact included those in *gyrA*, *parC*, *mgrA*, *saeR*, NWMN_RS05475, NWMN_RS13085, and *rarD* (Fig. S1A). Mutations in *gyrA* and *parC*, located within the quinolone resistance-determining region (QRDR), are known to confer high-level CIP resistance (43); R-CEF carried notable mutations in *gdpP*, *saeR*, *fmtA*, NWMN_RS04050, and NWMN_RS10470, most of which were missense mutations (Fig. S1B); R-CC, with the highest mutational burden, featured alterations in *gyrA*, *gdpP*, *rpoC*, *mgrA*, *saeR*, and *qoxA* (Fig. S1C). A significant reduction in hemolytic activity was observed for all three resistant strains in hemolysis assays (Fig. 1B and Fig. S1D). To assess potential cross-resistance, we determined MICs for a panel of ten additional antibiotics representing distinct classes: tetracycline (TET), cefoxitin (CFX), vancomycin (VAN), erythromycin (ERY), chloramphenicol (CHL), amikacin (AMK), clindamycin (CLIDM), nalidixic acid (NAL), amoxicillin (AMX), and carbenicillin (CAR). Compared with the wild-type strain, R-CEF and R-CC displayed a twofold reduction in MIC to TET, as well as eight-and twofold reductions to ERY and CHL, respectively. All three resistant strains showed a twofold decrease in MIC to VAN. In contrast, MICs to NAL increased fourfold in R-CIP and R-CEF, and eightfold in R-CC. Similarly, MICs to AMX rose twofold in R-CEF and fourfold in R-CC, while MICs to CAR increased fourfold in R-CEF and twofold in R-CC (Table 2). Survival assays were performed under AMX and NAL exposure further. R-CEF and R-CC survived better than the susceptible strain and R-CIP under AMX (Fig. 1C). All three resistant strains showed greater survival under NAL stress compared with the parent strain, in line with the MIC profiles (Fig. 1D). These results indicate that these three passaged antibiotic-resistant bacteria differ from the susceptible bacteria in some phenotypic characteristics, mainly in antibiotic sensitivity and hemolysis ability.

**TABLE 1:** MICs (μg/mL) of NM, RCIP, RCEF, and RCC against CIP and CEF

	Newman	R-CIP	R-CEF	R-CC
CIP (μg/mL)	0.3125	40	\^*a*^	40
CEF (μg/mL)	0.625	\	80	80

^*a*^ “\” indicates that MIC testing was not performed.

**TABLE 2:** MICs (μg/mL) of four strains against different antibiotics

	Newman	R-CIP	R-CEF	R-CC
TET (μg/mL)	10	10	5	5
CFX (μg/mL)	10	10	20	10
VAN (μg/mL)	2.5	1.25	1.25	1.25
ERY (μg/mL)	2.5	2.5	0.3	0.3
CHL (μg/mL)	10	10	5	5
AMK (μg/mL)	1.25	1.25	1.25	1.25
CLIDM (μg/mL)	20	20	40	40
NAL (μg/mL)	40	160	160	320
AM (μg/mL)	1.25	1.25	2.5	5
CAR (μg/mL)	2.5	2.5	10	5

### Comparative metabolomics of antibiotic-resistant bacteria

**Fig 2:**
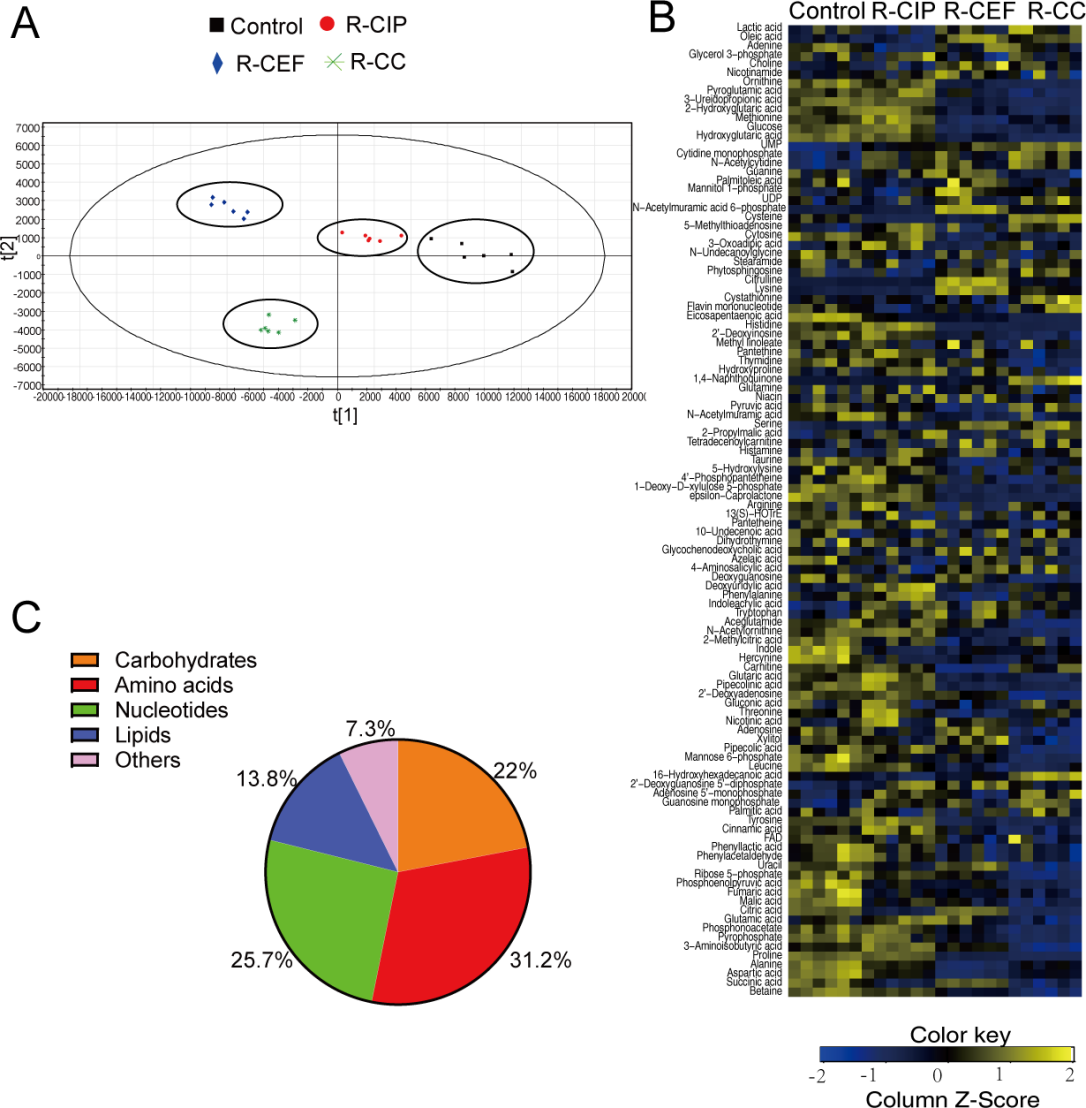
Comparative metabolic profiles of three drug-resistant strains and the control strain. (**A**) Principal component analysis (PCA) of drug-resistant and control bacterial strains. (**B**) Unsupervised hierarchical clustering heatmap (rows) of all metabolites. Blue and yellow indicate decreased and increased metabolite levels, respectively, scaled to the mean and standard deviation of row metabolite levels (see color scale). (**C**) Categories of all metabolites.

Given the known link between bacterial metabolism and antibiotic resistance, we performed comparative metabolomic profiling of the susceptible control strain (Newman) and the artificially selected resistant strains (R-CEF, R-CIP, R-CC) using LC-MS/MS. Six biological replicates were analyzed per group. The reproducibility of the measurements was evaluated by calculating pairwise correlation coefficients among six quality-control (QC) samples. All QC samples showed correlation coefficients above 0.82 (with values closer to 1 indicating higher reproducibility), confirming the technical reliability of the data set (Fig. S2A). Principal component analysis revealed clear separation between resistant strains and the susceptible control, reflecting the metabolic impact of antibiotic resistance. Within each group, biological replicates clustered tightly, further attesting to data reproducibility. It is noteworthy that the various drug-resistant bacteria can be clearly distinguished, with metabolic variations existing among different resistant strains (Fig. 2A). Following removal of ribitol and known artifact peaks, an unsupervised clustering analysis (ggplot2, R) was applied to generate a global metabolite heatmap, providing an overview of abundance changes across all strains (Fig. 2B). In total, 108 metabolites were identified and classified into five major categories: carbohydrates (22%), amino acids (31.2%), lipids (25.7%), nucleotides (13.8%), and others (7.3%) (Fig. 2C). The above results demonstrate that bacterial metabolic profiles undergo significant alterations upon acquiring drug resistance.

### Differential metabolomics of drug-resistant bacteria

**Fig 3:**
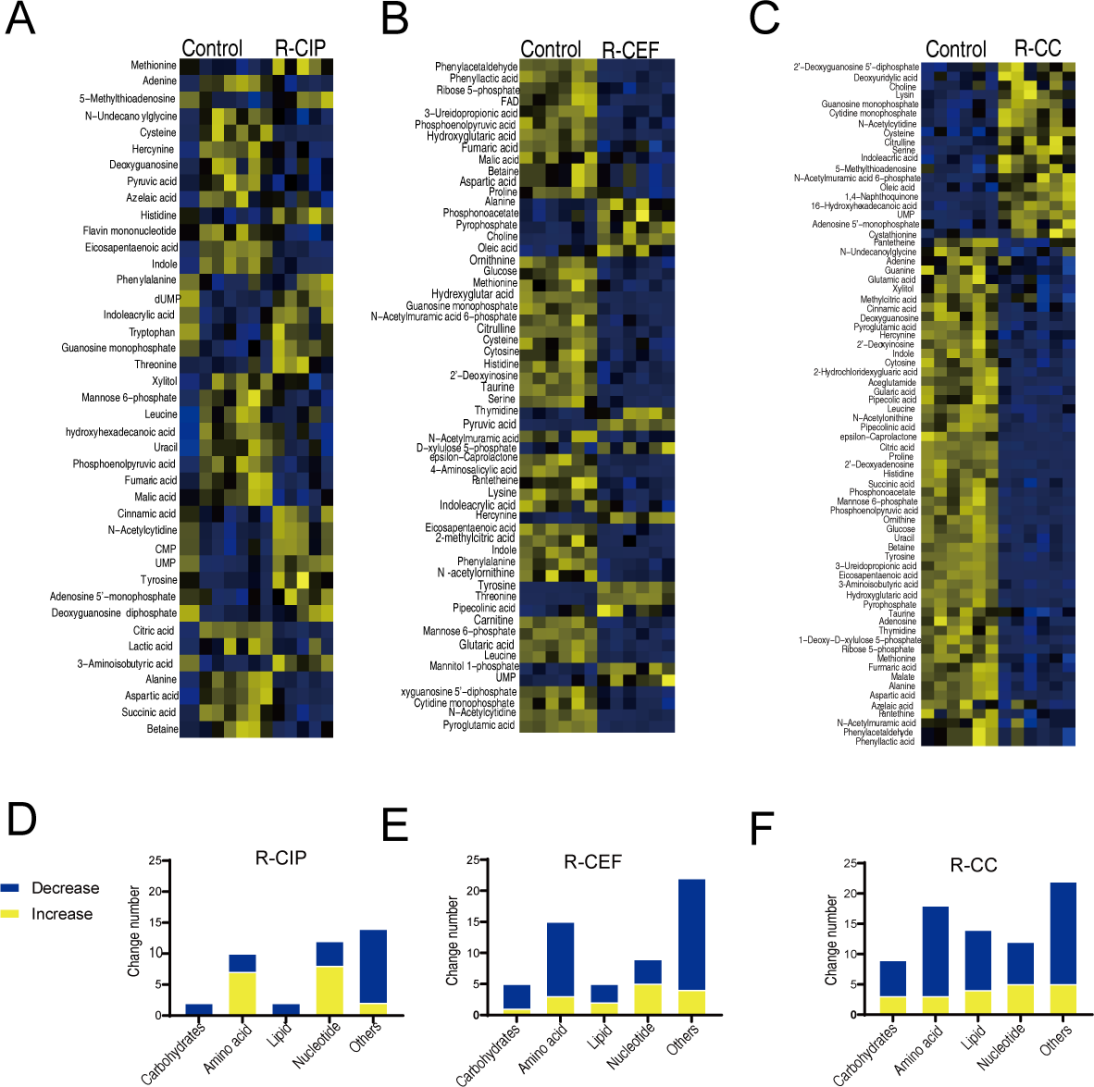
Distinct metabolic profiles of antibiotic-resistant strains. (**A–C**) Heatmaps displaying significantly altered metabolites in R-CIP, R-CEF, and R-CC strains, respectively. The color scale from blue to yellow represents low to high relative abundance. (**D–F**) Categorization of the differentially abundant metabolites. Metabolites highlighted in blue and yellow indicate increase and decrease, respectively.

To identify metabolic changes associated with antibiotic resistance, normalized metabolomic data from each resistant strain were compared with the susceptible control using the non-parametric Mann-Whitney U test. This analysis revealed 41 differentially abundant metabolites (DAMs) in R-CIP, 58 in R-CEF, and 74 in R-CC (Fig. 3A through C). The magnitude of change for each metabolite was expressed as a Z-score. Relative to the control, R-CIP contained 17 upregulated and 24 downregulated metabolites; R-CEF had 14 upregulated and 44 downregulated; R-CC showed 19 upregulated and 55 downregulated metabolites (Fig. S2B through D). This pattern indicates broad metabolic reprogramming in all resistant strains. Twenty-one DAMs were shared by all three resistant strains. Overlap was minimal between R-CIP and the other two strains, whereas R-CEF and R-CC shared the majority of their altered metabolites, most of which were downregulated (Fig. S2E through G). When classified by chemical category, several carbohydrates involved in central carbon metabolism were consistently downregulated in R-CIP, R-CEF, and R-CC (Fig. 3D through F). Together, these data illustrate both common and strain-specific metabolic adaptations in *S. aureus* following the acquisition of antibiotic resistance.

### Pathway enrichment analysis

**Fig 4:**
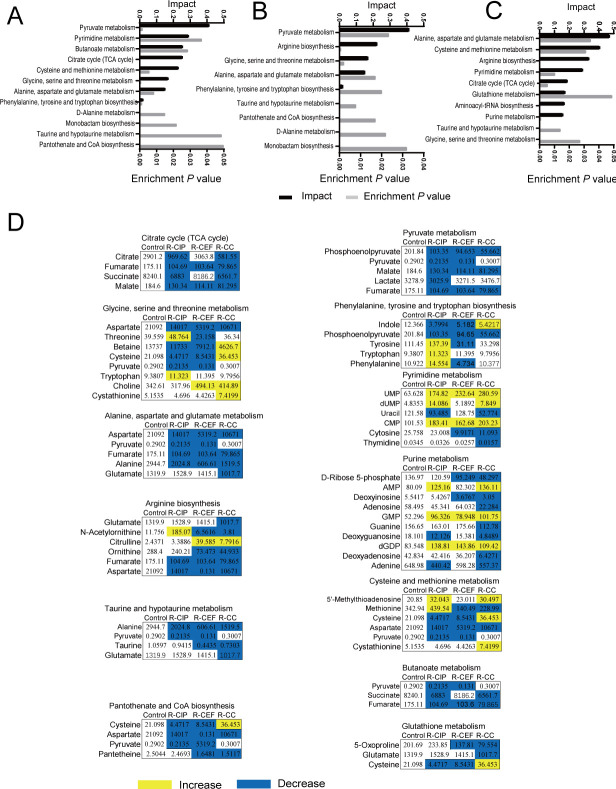
Pathway enrichment analysis of differential metabolites. (**A–C**) Pathway enrichment analysis was performed using the online platform MetaboAnalyst 5.0, with results shown for R-CIP, R-CEF, and R-CC in respective order. (**D**) Changes in metabolites within the metabolic pathways. Yellow indicates upregulated metabolites, while blue represents downregulated metabolites.

Pathway enrichment analysis was performed to map discrete metabolite changes to broader biological functions, thereby revealing systemic metabolic reprogramming under antibiotic stress and overcoming the interpretive limitations of isolated metabolite data. This analysis identified 12, 9, and 10 significantly perturbed pathways in R-CIP, R-CEF, and R-CC, respectively (Fig. 4A through C). Notably, three pathways were consistently enriched across all resistant strains: glycine, serine and threonine metabolism; alanine, aspartate and glutamate metabolism; and taurine and hypotaurine metabolism. This commonality suggests a convergent evolutionary strategy supporting resistance. Strain-specific pathway alterations were also observed: butanoate metabolism was uniquely altered in R-CIP, while glutamine metabolism and purine metabolism were specific to R-CC. No pathway was exclusively enriched in R-CEF. Glutamine metabolism and purine metabolism are metabolic pathways unique to R-CC. To assess whether enriched pathways were activated or suppressed, metabolite changes within each pathway were examined. In the commonly enriched alanine, aspartate, and glutamate metabolism pathway, nearly all metabolites were downregulated (Fig. 4D). Given the tight coupling between amino acid metabolism and energy production, this pattern likely reflects impaired energy metabolism. Similarly, metabolites in the TCA cycle—significantly enriched in R-CIP and R-CC—were markedly decreased, as were those in pyruvate metabolism, which was enriched in R-CIP and R-CEF. Although these two energy-related pathways were not simultaneously enriched across all three strains, their consistent metabolite declines strongly point to disrupted energy metabolism as a core resistance mechanism. Pathway-specific patterns further highlighted divergent adaptations among the resistant strains. In glycine, serine, and threonine metabolism, most metabolites were reduced in R-CIP and R-CEF but elevated in R-CC. Conversely, in aminoacyl-tRNA biosynthesis, metabolite levels increased in R-CIP but decreased in R-CEF and R-CC. All metabolites in pyruvate metabolism and pantothenate/CoA biosynthesis were downregulated in R-CIP and R-CEF. Arginine biosynthesis metabolites decreased predominantly in R-CEF and R-CC, while purine metabolism metabolites were uniformly down in R-CC. These findings illustrate that although each strain follows distinct adaptive routes at the pathway level, they converge on a common phenotype: broad impairment of energy metabolism.

### Antibiotic-induced biomarkers

**Fig 5:**
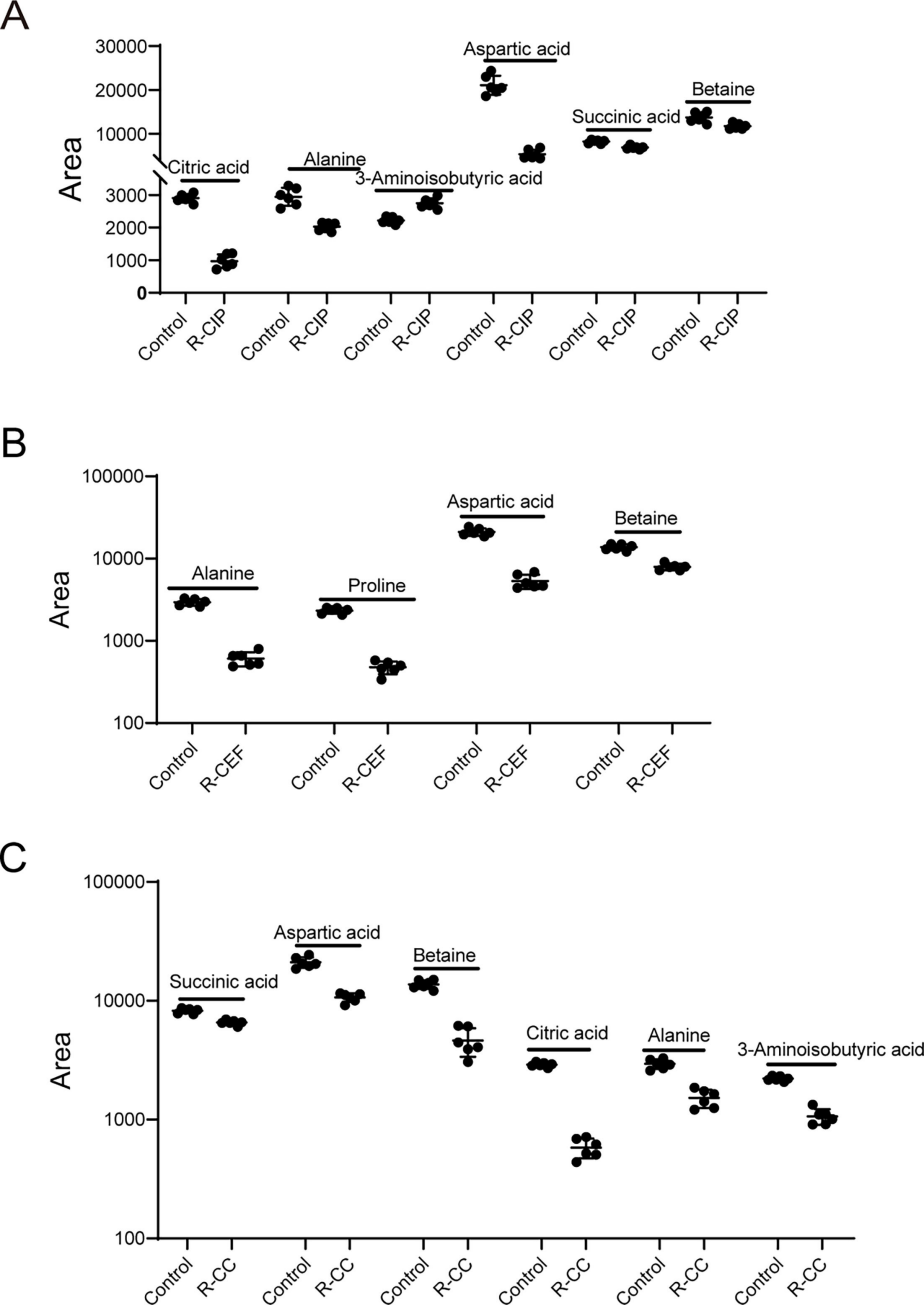
Biomarker candidate identification across resistant strains (R-CIP, R-CEF, R-CC).**(A–C)** Visualization of candidate biomarker distribution in the S-plot scatter diagram. R-CIP/Control (**A**), R-CEF (**B**), and R-CC/Control (**C**).

To explore sample patterns in the metabolome and identify key biomarkers, OPLS-DA was employed. The first predictive component, *t* (1), clearly separated the control from the resistant strains (Fig. S3A). For biomarker selection, an S-plot was generated, with metabolites meeting the thresholds (absolute covariance *P*|1| ≥ 0.05 and absolute correlation coefficient |*P*(corr)| ≥ 0.5) highlighted as red triangles. This analysis identified 6, 4, and 6 significant biomarkers in the R-CIP, R-CEF, and R-CC groups, respectively. Alanine, aspartate, and betaine were common to all three resistant strains. Citrate, succinate, and 3-aminobutyric acid were specifically altered in both R-CIP and R-CC (Fig. S3B). Compared with the control, the levels of alanine, aspartic acid, and betaine were decreased in all three resistant strains. In R-CEF and R-CC, citric acid and succinic acid were also reduced. Conversely, 3-aminobutyric acid was markedly elevated in R-CIP (Fig. 5A through C). Previous studies have shown that exogenous alanine enhances the bactericidal efficiency mediated by antimicrobial agents by increasing TCA cycle flux and inducing bacterial metabolic reprogramming (28, 35, 44). While intracellular alanine depletion conversely reduces flux through central carbon metabolism. Furthermore, the concurrent decrease in citric acid and succinic acid, two metabolites identified as shared biomarkers in R-CIP and R-CC. These results indicate that a downregulation of central carbon and energy metabolism represents a characteristic metabolic state associated with antibiotic resistance.

### The central carbon and energy metabolism in R-CIP, R-CEF, and R-CC are downregulated

**Fig 6:**
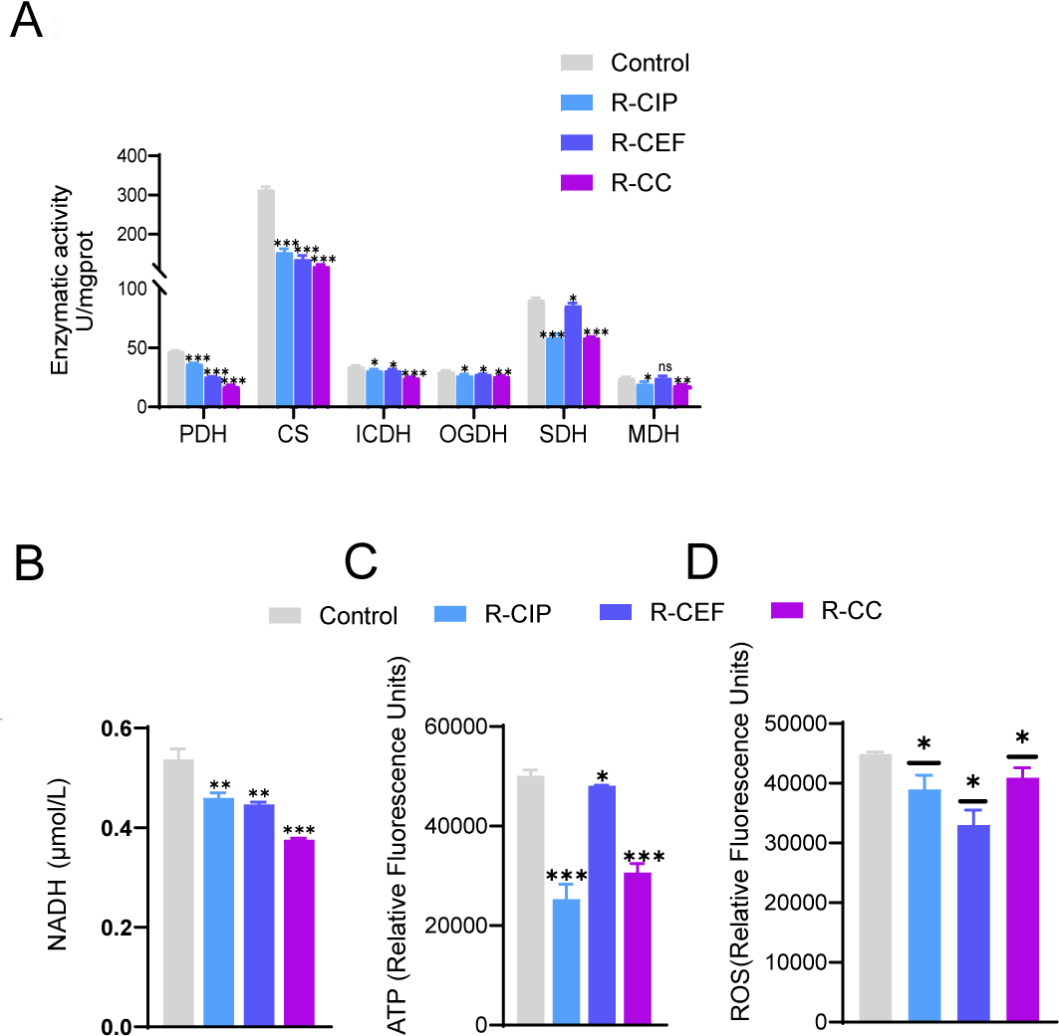
Functional validation of metabolic perturbations in drug-resistant strains. (**A**) Assay of the activities of pyruvate dehydrogenase (PDH), citrate synthase (CS), isocitrate dehydrogenase (ICDH), α-ketoglutarate dehydrogenase (OGDH), succinate dehydrogenase (SDH), and malate dehydrogenase (MDH). (**B**) Intracellular NADH levels. (**C**) ATP production quantified by luciferase assay. (**D**) ROS levels measured using DCFH-DA probe. (The results were shown as mean ± SEM, and the significant difference was determined by two-tailed Student’s *t*-test, ns indicated no significant difference, *P < 0.05, **P < 0.01, ***P < 0.001).

To visualize system-wide metabolic changes induced by antibiotic resistance, we generated global metabolic flux maps for the susceptible control and the resistant strains (R-CIP, R-CEF, R-CC) using the iPath interactive platform. As shown in Fig. S4A through C, all three resistant strains exhibited widespread metabolic suppression compared with the control. This was particularly pronounced in pathways central to bacterial growth and energetics, including central carbon metabolism, energy production, and amino acid biosynthesis. The consistent downregulation of these core metabolic processes underscores a fundamental rewiring of bacterial physiology associated with the acquired resistant phenotype. To functionally validate the observed metabolic alterations, we measured the activities of key metabolic enzymes. These included PDH (the key enzyme linking glycolysis to the TCA cycle), along with the rate-limiting TCA cycle enzymes CS, ICDH, and OGDH, as well as the related enzymes SDH and MDH. The activities of all assessed enzymes were significantly reduced in the resistant strains compared with the control, with the exception of MDH activity in the R-CEF group, which remained unchanged (Fig. 6A). The decrease was most pronounced in the dual-resistant R-CC strain. This concerted suppression of energy metabolism in the resistant strains resulted in a marked decrease in NADH production (Fig. 6B) and was accompanied by significantly diminished ATP production across all resistant strains (Fig. 6C). Consistent with impaired electron transport chain activity, ROS generation was also significantly lower in the resistant strains than in the susceptible control (Fig. 6D). These findings indicate that the suppression of central carbon and energy metabolism via metabolic reprogramming enables *S. aureus* to develop resistance to CIP and CEF.

### Intermediates of central carbon and energy metabolism can potentiate the killing efficacy of antibiotics

**Fig 7:**
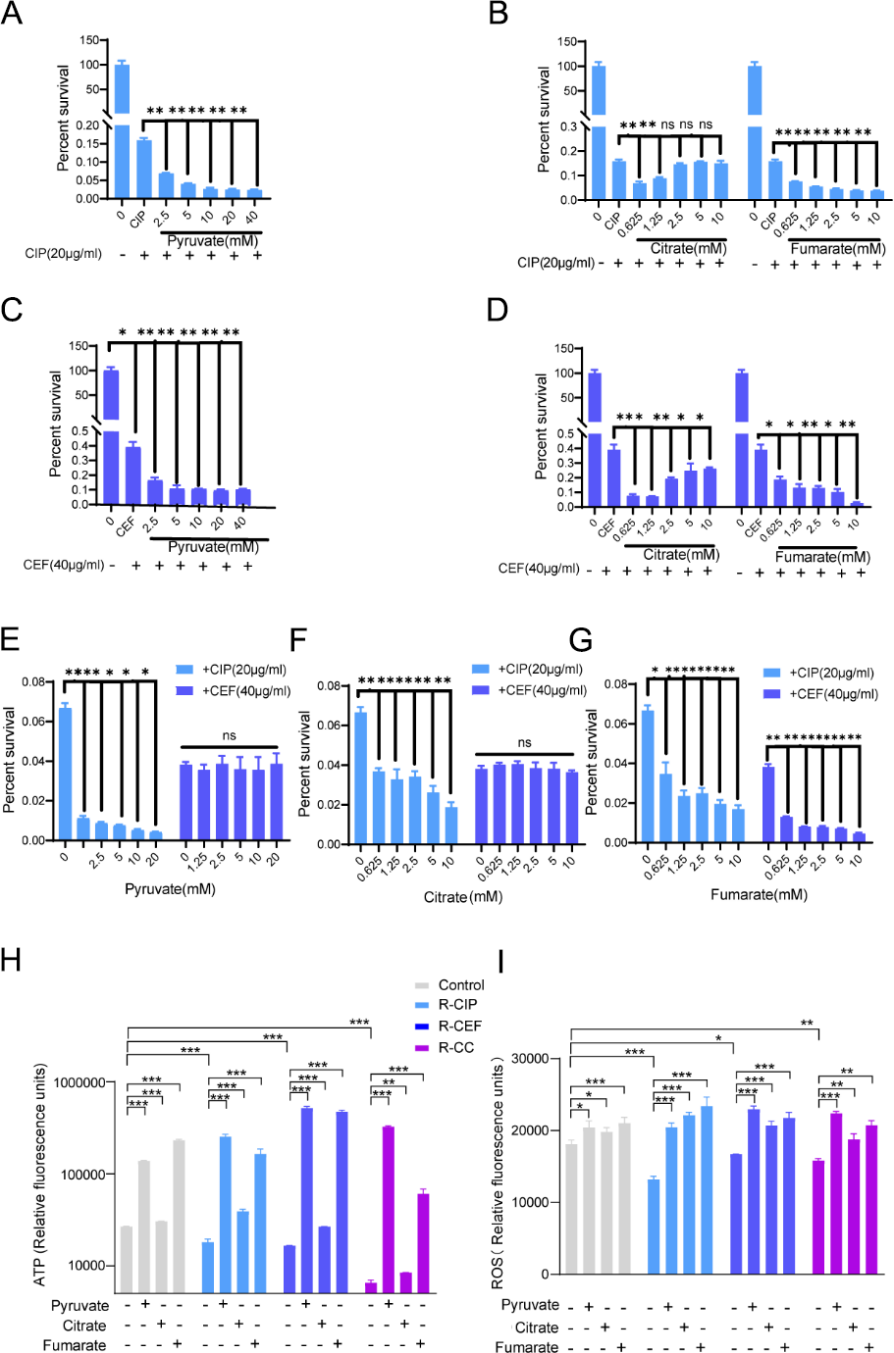
Exogenous metabolites restore antibiotic efficacy against resistant *S. aureus*. (**A and B**) Survival rate of the R-CIP strain in the presence of CIP combined with varying concentrations of pyruvate, citrate, or fumarate. (**C and D**) R-CEF survival under combinatorial treatment of CEF with pyruvate, citrate, or fumarate at indicated concentrations. (**E–G**) R-CC survival under exposure to CIP or CEF and various concentrations of pyruvate, citrate, or fumarate. (**H and I**) Measurements of ATP (**H**) and ROS levels (**I**) in the four strains upon supplementation with pyruvate, citrate, or fumarate. (The results were shown as mean ± SEM, and the significant difference was determined by two-tailed Student’s *t*-test, ns indicated no significant difference, *P < 0.05, **P < 0.01, ***P < 0.001.)

To validate the role of suppressed energy metabolism in conferring resistance, we developed a therapeutic strategy based on our pathway enrichment and OPLS-DA findings. This model employed co-treatment with antibiotics and key metabolites pyruvate (glycolysis), citrate, and fumarate (TCA cycle). Metabolite concentrations were selected within a range that did not affect bacterial viability when given alone (Fig. S5). Against R-CIP, pyruvate and fumarate enhanced ciprofloxacin (CIP) killing in a concentration-dependent manner, with maximum potentiation of 6.8- and 3.9-fold, respectively (Fig. 7A and B). Citrate improved CIP activity only at lower concentrations; higher concentrations reduced the effect. For R-CEF, pyruvate and fumarate increased cefazolin (CEF) efficacy up to 3.9- and 14-fold, respectively, also in a concentration-dependent manner (Fig. 7C and D). Citrate similarly enhanced killing, primarily at lower concentrations. In the dual-resistant strain R-CC, pyruvate and citrate markedly potentiated CIP (up to 16- and 3.5-fold, respectively) but did not improve CEF activity (Fig. 7E and F). Fumarate, however, enhanced both CIP and CEF against R-CC, showing concentration-dependent increases of up to four- and eightfold, respectively (Fig. 7G). Pyruvate, citrate, and fumarate are important intermediates in energy metabolism. Therefore, to further analyze how these metabolites affect the bactericidal efficacy of antibiotics, we measured the levels of ATP, a key product of energy metabolism. The results showed that exogenous addition of these metabolites significantly increased ATP levels in all four bacterial strains to varying degrees (Fig. 7H). Additionally, they also significantly elevated ROS levels in the bacteria (Fig. 7I). Collectively, these results indicate that despite a conserved strategy of metabolic suppression, resensitization effects are strain-dependent. Crucially, augmenting flux through central metabolic pathways represents a viable strategy to restore antibiotic efficacy in resistant populations.

## DISCUSSION

The relentless spread of drug-resistant *S. aureus* continues to erode the efficacy of existing antimicrobial defenses (44). A deeper understanding of the mechanisms that underpin this resistance is therefore essential for developing more effective therapeutic strategies (45). Despite the extensive clinical use of CEF and CIP against *S. aureus*, the escalating problem of drug resistance threatens their utility. A critical obstacle to addressing this challenge is the limited understanding of the pathogen’s metabolic adaptations to these antibiotics, a key area that this study aims to investigate. Our integrated metabolomic and functional analyses revealed a convergent evolution in the metabolic adaptation of *S. aureus* to both CEF and CIP. Although these antibiotics target distinct cellular processes—cell wall synthesis and DNA replication, respectively—resistance to both was linked to a suppression of central carbon metabolism. This indicates that the bacterial response to antibiotic stress involves a fundamental physiological reprogramming that extends beyond specific drug-target interactions.

Metabolomics has emerged as a key methodology for elucidating the mechanisms of bacterial antibiotic resistance by providing a direct functional readout of the cellular state (46). Unlike conventional approaches, it provides a global profile of metabolites, which are integral to all stages of the bacterial life cycle. Because shifts in key metabolite pools can critically alter antibiotic susceptibility, metabolomics is uniquely positioned to detect these functionally meaningful changes, revealing the physiological state that supports resistance (27, 47–50). Comparative metabolomics of drug-sensitive and -resistant bacteria has identified key biomarkers and pathways for reversing resistance. For example, in carbapenem-resistant *E. coli,* downregulated pyruvate formate-lyase (PFL) impaired micronomicin efficacy, while exogenous pyruvate and formate restored PFL activity, increasing membrane permeability and drug susceptibility (50). Similarly, in mcr-1-harboring bacteria, reduced succinyl-CoA shunts metabolism toward glycerophospholipid synthesis, promoting colistin resistance; succinate supplementation reverses this shift and restores sensitivity (51).

Growth phenotyping highlighted a fitness cost linked to cefazolin resistance: R-CEF and R-CC strains grew more slowly and reached lower maximum biomass than the susceptible parent. In contrast, R-CIP showed no such growth defect, implying that the metabolic adaptations underlying CIP resistance are less detrimental to proliferation. This divergence underscores how metabolic trade-offs during resistance acquisition are antibiotic-specific. this observed growth phenotype for CIP-resistant *S. aureus* is consistent with previous findings reported by Liu et al. (52). Metabolite profiling revealed widespread downregulation across resistant strains: 24 of 41 altered metabolites in R-CIP, 44 of 58 in R-CEF, and 55 of 74 in R-CC were reduced, with glycolysis and TCA cycle intermediates notably decreased. This pattern reflects progressive metabolic remodeling under sustained antibiotic selection. The dual-resistant R-CC strain displayed the most extensive alterations, suggesting that combined drug pressure imposes the most severe metabolic reprogramming—likely corresponding to a greater adaptive challenge. Pathway analysis identified both antibiotic-specific and shared perturbations. Pyrimidine and purine metabolism were co-enriched in R-CIP and R-CC; arginine metabolism was altered in R-CEF and R-CC. Three pathways were consistently dysregulated in all resistant strains: glycine, serine, threonine; alanine, aspartate, glutamate, and taurine and hypotaurine metabolism. The concordance between pathway enrichment (showing impaired energy metabolism) and the reduction in key biomarkers (e.g., alanine, citrate, succinate) in the OPLS-DA model points consistently to a globally attenuated metabolic state. iPath-based global analysis further confirmed that downregulation of central carbon and energy metabolism is a common feature of all three resistant strains. Through experimental validation, we found that the activities of PDH and key enzymes in the TCA cycle were reduced in all resistant strains, leading to decreased production of ATP and NADH. This phenomenon was most pronounced in the R-CC strain. This “energy crisis” not only limits growth but also likely diminishes antibiotic efficacy by reducing target activity and ROS generation. While some controversy persists, the prevailing view suggests that elevated ROS levels contribute to bacterial killing, whereas diminished ROS generation is linked to the development of antibiotic resistance (53–55). Both CEF (a β-lactam) and CIP (a fluoroquinolone) are considered bactericidal and have been shown to stimulate free-radical production, which contributes to their killing activity (56–58). Thus, the downregulation of central carbon metabolism appears to be a strategic trade-off in *S. aureus*, enhancing survival under antibiotic stress by reducing metabolic activity and concomitant ROS generation.

There is increasing evidence that central carbon and energy metabolism is a crucial determinant of antibiotic susceptibility. For example, Zhang et al. demonstrated that gentamycin resistance in *V. alginolyticus* involves suppression of glycolysis and pyruvate metabolism, leading to reduced ATP, NADH, and ROS production—a key mechanism underlying resistance (59). Similarly, a study by Zalis and colleagues showed that disruption of the TCA cycle in *S. aureus*, achieved through deletion of genes such as *gltA*, forces the bacterium into a low-energy state with diminished ATP output and increased drug tolerance (60). Furthermore, the downregulation of central carbon and energy metabolism in *S. aureus* results in low intracellular ATP, which in turn promotes persister cell formation or enhances both antibiotic tolerance and resistance (61–63). Previous studies have identified pyruvate carboxylase (PycA) as a central metabolic hub, which is intrinsically linked to its antimicrobial resistance and food system persistence (64). Our previous studies have found that downregulation of central carbon metabolism and reduced energy production are closely related to the development of gentamycin resistance in *S. aureus* (40). Many studies have found that tricarboxylic acid cycle disorders and regulation of nucleotide- and amino acid-metabolic pathways lead to ATP depletion, thereby promoting the formation of persister cells of *S. aureus* and enhancing resistance to multiple antimicrobial drugs (62, 65, 66).

Pathway enrichment analysis revealed differential regulation of glycine, serine, and threonine metabolism in drug-resistant strains: this pathway was downregulated in R-CIP and R-CEF strains but upregulated in R-CC strains. In R-CC, purine metabolism is downregulated, while glycine, serine, and threonine metabolism serve as the primary source of one-carbon units, which are key precursors for nucleotides. Therefore, under dual-drug pressure, R-CC may enhance nucleotide synthesis through this mechanism. It reflects the plasticity and complexity exhibited by bacterial metabolic networks when facing different environmental challenges. However, the specific mechanisms involved require further validation. Our previous studies have confirmed that exogenous supplementation of glycine and threonine enhances the bactericidal effect of gentamycin against MRSA (67). Similarly, Cheng et al. reported that glycine uptake can promote TCA cycle flux, aiding serum killing capacity by increasing NADH levels and membrane potential (68). Therefore, the downregulation of this pathway in R-CIP and R-CEF strains may represent a conserved resistance strategy—limiting the entry of these amino acids into the TCA cycle to weaken energy metabolism. By contrast, its upregulation in R-CC suggests a distinct, strain-specific metabolic adaptation, possibly to compensate for other deficits or to engage an alternative resistance route; this divergence merits further study. Consistent with the observed enzymatic activities, the attenuation of alanine, aspartate, and glutamate metabolism in all three resistant strains likely reduces the flux of key substrates (e.g., pyruvate, citrate) into the TCA cycle. This restriction directly contributes to diminished energy production, thereby helping to maintain the bacteria in a low-energy state that is less susceptible to antibiotic action (28, 69). Consistent with this hypothesis, our findings confirm that elevating the concentration of key substrates to drive metabolic flux into the energy cycle effectively enhances antibiotic efficacy against the resistant strains (Fig. 7). Notably, the therapeutic application of metabolite supplementation warrants careful consideration. *In vivo*, exogenous metabolites may be utilized as nutrients by pathogens, and their bioavailability and local concentration pose challenges. Future studies can focus on targeted delivery systems, to enhance therapeutic specificity and safety.

Previous studies have highlighted metabolic modulation as a strategy to enhance antibiotic efficacy against MRSA. For example, Li et al. identified the biomarker valine promotes MRSA membrane permeability by activating the TCA cycle and increasing proton motive force, thereby boosting the activity of cefoperazone-sulbactam (69). Similarly, fatty acids have been shown against MRSA, demonstrating their ability to enhance antibacterial and anti-biofilm activities (70). Correspondingly, a recent study demonstrates that combining 5-fluorouracil with fosfomycin disrupts pyrimidine metabolism, leading to membrane damage, PMF dissipation, elevated ATP synthesis, and ROS accumulation, which together enhance antibacterial activity against MRSA (71). The bacterial energy metabolic state can exert a broad influence on various cellular pathways, thereby contributing to antibiotic resistance. Our previous research demonstrated that *S. aureus*, under ampicillin and gentamycin stress, upregulates arginine biosynthesis. This metabolic shift facilitates increased nitric oxide production, which subsequently neutralizes ROS and mediates the development of resistance (39). Evidence from diverse bacteria, such as *E. tarda* where ciprofloxacin resistance involves energy cycle disruption and altered membrane permeability, further substantiates that metabolic reprogramming is a central strategy in the evolution of antibiotic resistance (36). The relationship between bacterial metabolism and physiology is complex. Compared with the wild-type strain, R-CEF and R-CC showed slower growth and lower ATP levels, suggesting an elevated metabolic burden to maintain antibiotic resistance. In contrast, R-CIP maintained ciprofloxacin resistance and a high growth rate, yet also exhibited reduced ATP levels. This may indicate higher energy utilization efficiency or other metabolic adaptations, the molecular basis of which warrants further investigation.

In conclusion, antibiotic resistance in *S. aureus* is driven by a conserved metabolic downregulation of central carbon and energy pathways, leading to a low-energy state that reduces antibiotic susceptibility. This metabolic suppression, observed in strains resistant to CIP and CEF, diminishes ATP, NADH, and ROS production, thereby limiting drug efficacy. Importantly, exogenous supplementation of key metabolic intermediates (e.g., pyruvate, citrate, fumarate) can reprogram resistant cells, restore energy flux, and resensitize them to antibiotics. These findings reveal metabolic reprogramming as a targetable vulnerability for overcoming resistance.

## Data Availability

Data will be made available on request. The LC-MS/MS metabolomics data are available in FigShare (https://doi.org/10.6084/m9.figshare.31932984).

## SUPPLEMENTAL MATERIAL

Supplemental material spectrum.00020-26-s0001.docx Fig. S1 to S5; Tables S1 to S3.

ASM does not own the copyrights to Supplemental Material that may be linked to, or accessed through, an article. The authors have granted ASM a non-exclusive, world-wide license to publish the Supplemental Material files. Please contact the corresponding author directly for reuse.
