# Supplementary material for: Metabolic adaptations in antibiotic-resistant Staphylococcus aureus: understanding resistance mechanisms and enhancing antibiotic efficacy
Source: Microbiol Spectr. 2026 Jun 29;14(8):e00020-26. doi: 10.1128/spectrum.00020-26 (PMC13435697; doi:10.1128/spectrum.00020-26)
Supplement: Supplementary file 1 [file spectrum.00020-26-s0001.docx]

**Supplementary Figures and Tables**

**Supplementary FIG 1** Whole-genome sequencing and hemolysis assay of drug-resistant bacteria. (A-C) Comparative genomic analysis of R-CIP (A), R-CEF (B), R-CC (C) with Newman strains, respectively. (D) Hemolysis assay (ddH_2_O as positive control, saline as negative control).

**Supplementary FIG 2** Analysis of quality control and differential metabolites. (A) QC correlation matrix plot. (B-D) Z-score plots for each resistant strain. (E) All differential metabolites. (F) Upregulated differential metabolites. (G) Downregulated differential metabolites.

**Supplementary FIG 3** OPLS-DA-based comparative analysis and biomarker screening. (A) OPLS-DA analysis of R-CIP, R-CEF, and R-CC. Each dot represents the biological replicate analysis of samples in the plot. (B) The S-plot derived from OPLS-DA modeling visualized metabolic features as triangles, with potential biomarker candidates (highlighted in red) selected based on statistical thresholds (|p(covariance)| ≥ 0.05 and |p(correlation)| ≥ 0.5). R-CIP (left), R-CEF (middle), R-CC (right).

**Supplementary FIG 4** iPath analysis of global metabolic changes in antibiotic-resistant bacteria. (A-C) iPath-based global metabolic maps illustrating pathway alterations in (A) R-CIP, (B) R-CEF, and (C) R-CC groups. Metabolite changes are color-coded: red for up-regulation, blue for down-regulation.

**Supplementary FIG 5** Effects of individual metabolites on three resistant strains. (A-C) Viability of R‑CIP, R‑CEF, and R‑CC, respectively, in the presence of increasing concentrations of pyruvate, citrate, or fumarate.

**Table S1** Mutation details in R‑CIP

| Gene name | Location | Mutation | Variantion type | Amino acid substitution | Function |
| --- | --- | --- | --- | --- | --- |
| *gyrA* | 7262 | missense_variant | c.251C>T | p.Ser84Leu | DNA gyrase subunit A |
| *mgrA* | 737121 | frameshift_variant | c.79delT | p.Ser27fs | HTH-type transcriptional regulator MgrA |
| *saeR* | 758252 | frameshift_variant | c.109dupG | p.Ala37fs | response regulator transcription factor SaeR |
| *NWMN_RS05475* | 1083348 | frameshift_variant | c.201_205dupCGAAG | p.Asp69fs | YlaN family protein |
| *parC* | 1394401 | missense_variant | c.250G>A | p.Glu84Lys | DNA topoisomerase IV subunit A |
| *budA* | 2353160 | upstream_gene_variant | c.-4403_-4402insT | N.A | acetolactate decarboxylase |
| *NWMN_RS13085* | 2498278 | frameshift_variant | c.476_480dupCAGCA | p.Leu161fs | NAD(P)/FAD-dependent oxidoreductase |
| *rarD* | *2861275* | *frameshift_variant* | *c.302dupG* | *p.His102fs* | *EamA family transporter RarD* |

**Table S2** Mutation details in R‑CEF

| Gene name | Location | Mutation | Variantion type | Amino acid substitution | Function |
| --- | --- | --- | --- | --- | --- |
| *gdpP* | 19577 | missense_variant | c.1253A>G | p.Asp418Gly | cyclic-di-AMP phosphodiesterase GdpP |
| *pbp4* | 693489 | upstream_gene_variant | c.-377_-376insA | N.A | penicillin-binding protein PBP4 |
| *saeR* | 757812 | missense_variant | c.550T>C | p.Tyr184His | response regulator transcription factor SaeR |
| *NWMN_RS04050* | 802359 | missense_variant | c.491G>A | p.Gly164Glu | glycosyltransferase family 4 protein |
| *fmtA* | 1028488 | missense_variant | c.850G>A | p.Gly284Ser | teichoic acid D-Ala esterase FmtA |
| *NWMN_RS10470* | 2029908 | missense_variant | c.961T>G | p.Phe321Val | sensor histidine kinase VraS |

**Table S3** Mutation details in R‑CC

| Gene name | Location | Mutation | Variantion type | Amino acid substitution | Function |
| --- | --- | --- | --- | --- | --- |
| *gyrA* | 7262 | missense_variant | c.251C>T | p.Ser84Leu | DNA gyrase subunit A |
| *gdpP* | 19291 | frameshift_variant | c.968dupT | p.Ile324fs | cyclic-di-AMP phosphodiesterase GdpP |
| *rpoC* | 581751 | missense_variant | c.1960G>T | p.Gly654Cys | DNA-directed RNA polymerase subunit beta' |
| *rpoC* | 581752 | missense_variant | c.1961G>T | p.Gly654Val | DNA-directed RNA polymerase subunit beta' |
| *mgrA* | 737121 | frameshift_variant | c.79delT | p.Ser27fs | HTH-type transcriptional regulator MgrA |
| *saeR* | 758252 | frameshift_variant | c.109dupG | p.Ala37fs | response regulator transcription factor SaeR |
| *qoxA* | 1032453 | frameshift_variant | c.886_887insG | p.Lys296fs | cytochrome aa3 quinol oxidase subunit II |
| *qoxA* | 1032456 | missense_variant | c.884T>A | p.Phe295Tyr | cytochrome aa3 quinol oxidase subunit II |
| *qoxA* | 1032457 | missense_variant | c.883T>C | p.Phe295Leu | cytochrome aa3 quinol oxidase subunit II |
| *qoxA* | 1032462 | frameshift_variant | c.864_877delCACTAGTGAAGAAA | p.Phe288fs | cytochrome aa3 quinol oxidase subunit II |
| *qoxA* | 1032478 | frameshift_variant | c.861_862insGT | p.Phe288fs | cytochrome aa3 quinol oxidase subunit II |
| *qoxA* | 1032479 | frameshift_variant | c.853_860delGATCCAAA | p.Asp285fs | cytochrome aa3 quinol oxidase subunit II |
| *NWMN_RS05475* | 1083348 | frameshift_variant | c.201_205dupCGAAG | p.Asp69fs | YlaN family protein |
| *NWMN_RS06185* | 1195325 | missense_variant | c.1495C>G | p.His499Asp | penicillin-binding protein |
| *parC* | 1394401 | missense_variant | c.250G>A | p.Glu84Lys | DNA topoisomerase IV subunit A |
| *NWMN_RS07670* | 1526152 | missense_variant | c.1756A>G | p.Thr586Ala | transglycosylase domain-containing protein |
| *cdaA* | 2293456 | missense_variant | c.155T>C | p.Val52Ala | diadenylate cyclase CdaA |
| *budA* | 2353160 | upstream_gene_variant | c.-4403_-4402insT | N.A | acetolactate decarboxylase |
| *NWMN_RS13085* | 2498278 | frameshift_variant | c.476_480dupCAGCA | p.Leu161fs | NAD(P)/FAD-dependent oxidoreductase |
| *rarD* | 2861275 | frameshift_variant | c.302dupG | p.His102fs | EamA family transporter RarD |
